# The impact of group antenatal care on newborns: Results of a cluster randomized control trial in Eastern Region, Ghana

**DOI:** 10.1186/s12887-024-05225-9

**Published:** 2024-11-18

**Authors:** Veronica Apetorgbor, Elizabeth Awini, Bidisha Ghosh, Ruth Zielinski, Georgina Amankwah, Vida A. Kukula, Katherine James, John E.O. Williams, Jody R. Lori, Cheryl A. Moyer

**Affiliations:** 1 Dodowa Health Research Center, Ghana Health Service, Dodowa, Ghana; 2 University of Michigan School of Nursing, Ann Arbor, MI USA; 3 Regional Health Directorate, Ghana Health Service, Koforidua, Eastern Region Ghana; 4 Departments of Learning Health Sciences, Obstetrics & Gynecology, Health Management and Policy, University of Michigan, 1111 E. Catherine Street, 231 Victor Vaughan Bldg, Ann Arbor, MI 48109 USA

**Keywords:** Antenatal care, Group antenatal care, Prenatal care, Ghana, Sub-saharan Africa, Care seeking, Maternal morbidity, Neonatal morbidity, Maternal outcomes, Neonatal outcomes

## Abstract

**Background:**

Maternal recognition of neonatal danger signs following birth is a strong predictor of care-seeking for newborn illness, which increases the odds of newborn survival. However, research suggests that maternal knowledge of newborn danger signs is low. Similarly, maternal knowledge of optimal newborn care practices has also been shown to be low. Since both issues are typically addressed during antenatal care, this study sought to determine whether group antenatal care (G-ANC) could lead to improvements in maternal recognition of danger signs and knowledge of healthy newborn practices, as well as boosting postnatal care utilization.

**Methods:**

This cluster randomized controlled trial of G-ANC compared to routine individual antenatal care (I-ANC) was conducted at 14 health facilities in Ghana, West Africa, from July 2019 to July 2023. Facilities were randomized to intervention or control, and pregnant participants at each facility were recruited into groups and followed for the duration of their pregnancies. 1761 participants were recruited: 877 into G-ANC; 884 into I-ANC. Data collection occurred at enrollment (T0), 34 weeks’ gestation to 3 weeks postdelivery (T1) and 6–12 weeks postpartum (T2). Comparisons were made across groups and over time using logistic regression adjusted for clustering.

**Results:**

Overall, knowledge of newborn danger signs was significantly higher for women in G-ANC, both in aggregate (13-point scale) and for many of the individual items over time. Likewise, knowledge of what is needed to keep a newborn healthy was higher among women in G-ANC compared to I-ANC over time for the aggregate (7-point scale) and for many of the individual items. Women in G-ANC were less likely to report postnatal visits for themselves and their babies within 2 days of delivery than women in I-ANC, and there was no difference between groups regarding postnatal visits at one week or 6 weeks after birth.

**Conclusion:**

This study illustrates that group ANC significantly improves knowledge of newborn danger signs and healthy newborn practices when compared to routine care, suggesting that the impact of G-ANC extends beyond impacts on maternal health. Further research elucidating care pathways for ill newborns and maternal behaviors around healthy newborn practices is warranted.

**Trial registration:**

: ClinicalTrials.gov Identifier: NCT04033003, Registered: July 25, 2019 Protocol Available at: https://www.ncbi.nlm.nih.gov/pmc/articles/PMC9508671/.

## Introduction

Pregnancy and childbirth pose a significant risk to mothers and babies in low- and middle-income countries (LMICs). Nearly 300,000 women die each year from pregnancy-related causes each year [1], and 2.3 million babies die within a month of being born [2]. While Ghana has better health indicators than many of its West African neighbors, it faces significant challenges in improving maternal and newborn health [3, 4]. An average of 19 out of every 1,000 babies born each year in Ghana do not survive their first month of life [3]. 

Maternal recognition of neonatal danger signs following birth is a strong predictor of care-seeking for newborn illness [5], which increases the odds of newborn survival. However, research suggests that knowledge of newborn danger signs is low. In one study in West Africa, 95% of mothers could name only one newborn danger sign (fever), and fewer than 30% could name more than three [6]. Maternal knowledge of how to keep babies healthy has also been shown to be limited. In a recent study in Ethiopia, half of mothers had adequate knowledge of essential newborn care practices [7]. In addition, recognition of the importance of postnatal care for newborns is inconsistent, as evidenced by wide variability in postnatal care utilization throughout sub-Saharan Africa [8]. According to the 2022 Ghana Demographic Health Survey, only 52% of newborns had a postnatal health check after birth [9]. 

All of these issues – recognition of neonatal danger signs, understanding essential newborn care practices, and encouragement to seek postnatal care – are ideally addressed through routine antenatal care. Antenatal care is seen as one of the best ways to address poor outcomes among both mothers and babies, given repeated studies that have shown better maternal, fetal, and neonatal outcomes among pregnant patients who have had adequate antenatal care [10, 11]. Nonetheless, despite increasing rates of ANC uptake throughout sub-Saharan Africa, rates of maternal and newborn mortality have not improved as much as might be expected [1, 4]. Group antenatal care (G-ANC), or delivering care to women in gestationally-age matched small groups throughout the duration of their pregnancy, has been explored as a way to make ANC more effective. G-ANC has been linked to improved women’s health literacy [12, 13], antenatal care utilization [12, 14], birth preparedness [12], pregnancy related empowerment [15], facility-based delivery [16], health outcomes [17, 18], and postpartum planning [12]. Inconsistently, G-ANC has been linked to improved postnatal care uptake [16]. In high-resource settings, G-ANC has been linked to lower rates of preterm birth [19, 20] as well as a host of other positive outcomes [18, 21, 22]. Little research has explored the impact that G-ANC has on newborns. This manuscript sought to: (1) assess the impact of G-ANC on mothers’ recognition of newborn danger signs and recall of actions that can be taken to keep newborns healthy; and (2) determine if G-ANC impacted utilization of postnatal care for newborns within 48 h, at 6–7 days, or at 6 weeks after birth.

## Methods

### Study design and setting

We conducted a cluster randomized controlled trial at 14 health facilities in the Eastern Region of Ghana that compared G-ANC (grouping women by gestational age) to standard individual antenatal care. (ClinicalTrials.gov Identifier: NCT04033003, 25/07/2019; available at: DOI: 10.2196/40828) Facilities were selected across four districts (Nsawam, Yilo Krobo, Akwapim North, and Lower Manya Krobo) and were randomized using a matched pairs design. Each pair of facilities was similar in the number of deliveries per month and average gestational age at first antenatal visit, and within each pair, one facility was randomly assigned to intervention (G-ANC) and the other to control (I-ANC). In this region of Ghana, the total fertility rate was 3.1, births are relatively evenly split between urban (47.5%) and rural (52.5%) areas [23]. (According to the 2021 Population of Regions and Districts Report, the Eastern Region had 68,955 births in 2021, with a maternal mortality ratio of 317 per 100,000 and an infant mortality ratio of 33.9 per 1000 live births [23]. Full details of the methods are available elsewhere [24], and CONSORT 2010 guidelines were followed [25]. 

### Power and sample size calculations

As described in the previously published protocol [23], we calculated sample size based on three primary outcomes: changes in birth preparedness and complication readiness scores, the percent change in women obtaining maternal postpartum checkups, and newborns obtaining postnatal checkups within the first 2 days after birth. Our cluster randomized design meant that intraclass correlation coefficient – or the extent to which the effect of an intervention might differ across facilities – was included in our calculations. At 80% power and 0.05 significance, all three outcome measures required a sample size of approximately 100 women per facility, or 1400 women total. To account for potential attrition, we aimed to recruit 120 women per facility, or a total of 1680 [23]. 

### Recruitment of participants and informed consent

Eligibility for enrollment of the women included: [1] less than 20 weeks’ gestation at first ANC visit; [2] speaks Dangme, Ga, Akan, Ewe, or English; [3] over 15 years of age; and [4] not considered a high-risk pregnancy at the time of enrollment. At each facility, a midwife trained in the study protocol instructed women who were likely to be eligible to participate in the study to talk to the research assistant (RA) if they were interested in learning more about the study. Women who approached the RA were read a recruitment script, their eligibility was confirmed, and those willing to participate were taken through an informed consent procedure prior to baseline data collection.

### Intervention

The G-ANC model consisted of nine meetings, one was individual meeting followed by eight group interactive sessions throughout pregnancy. Upon enrollment, women were put into groups of 10-14 women based on similar gestational age. Women met with the midwife individually during their first visit, and subsequent visits included additional group meetings. Details of the G-ANC intervention are described in detail elsewhere [24], but in summary, group meetings lasted an average of 60–90 min and were facilitated by a trained midwife. Sessions were designed to use strategies such as storytelling, peer support, demonstration and teach-back to encourage a participatory and interactive approach to antenatal care. Topics addressed included such things as malaria prophylaxis, healthy habits during pregnancy, preparing for birth, recognizing danger signs during pregnancy, recognizing danger signs in newborns, and family planning. Participants were given an illustrated booklet to take home that addressed each topic covered in the group sessions, and they were encouraged to talk with their family members about what they learned. Fidelity to the G-ANC model was ensured through routine monitoring by both local trainers and the researchers, who collected and reviewed the tracking logs of the participating midwives and who observed group sessions and completed periodic fidelity checklists to ensure all aspects of the content and methodology were addressed. Results of the fidelity assessments have been published elsewhere [26]. 

Women in facilities randomized to the control group participated in routine, individual antenatal care. Routine ANC in Ghana is based on WHO guidelines and includes a recommended 8 visits with a healthcare provider during pregnancy, during which time the pregnant women receives standard screenings (e.g. blood pressure checks, urine tests, etc.), necessary interventions (e.g. malaria prophylaxis, folate supplementation, etc.), nutritional counseling, and education on pregnancy and childbirth. Danger signs for newborns are included in the educational component for mothers near the end of their pregnancies and are reiterated during postnatal care.

### Data collection tools

Multiple measures were used for data collection. In addition to an assessment of basic demographic characteristics (such as age, education, religion, parity, location of delivery), this study utilized an instrument previously used by JHPIEGO to assess maternal recognition of newborn danger signs [27]. The specific question related to newborn danger signs asked, “What are danger/warning signs for your newborn? These are signs that there may be a problem and you need to seek care from a health worker.” The options included: (1) too hot or too cold, (2) convulsions/fits, (3) little or no movement, (4) feeding poorly or not at all, (5) fast breathing or chest indrawing, (6) not passing urine and/or stool, (7) umbilical cord stump is bleeding / has a foul odor / has redness around it, (8) very small baby born at home, (9) yellow skin, eyes, palms or soles of feet, (10) red swollen eyes with pus, 11) persistent vomiting, 12) diarrhea, 13) weak cry, and don’t know. These items were analyzed separately (provided there was sufficient N per cell) and then combined into a 13-point scale for analysis. Measurement also included an item that asked mothers what they thought was important to keep their newborns healthy after birth. Responses included: (1) keep head and body covered, (2) feed only breastmilk for 6 months, (3) breastfeed often / whenever the baby wants, (4) keep cord stump clean and dry, (5) wash hands with soap and water at all times before handling baby, (6) have baby immunized, (7) go for post-natal visit, and don’t know. These items were analyzed separately (provided there was sufficient N per cell) and then combined into a 7-point scale for analysis. Data were collected on newborn care upon enrollment and again at 34 weeks’ gestation to 3 weeks postdelivery.

Service utilization data (including number and timing of postnatal care visits) was collected using a standardized questionnaire given to participating mothers at 6–12 weeks post-partum (T2). This included asking about whether postnatal visits were obtained within 48 h, at 6–7 days, and at 6 weeks (yes/no).

### Data collection and statistical analyses

Trained local research assistants (RAs) collected baseline demographic data upon enrollment in the study following informed consent (T0). Knowledge of danger signs for both mothers and babies were collected at enrollment (T0) and again at the end of pregnancy, a data collection window that corresponded to 34 weeks’ gestation to 3 weeks postdelivery (T1). Postnatal visit data were collected at 6 weeks post-partum (T2). All data were collected using encrypted and password-protected tablets, and entered into REDCap, a secure web-based application for data collection and database management.

SAS 9.4 was used for data management while Stata 18.0 was used to conduct statistical analyses. Data collected on knowledge of danger sings and newborn care at T0 and T1 for the control and intervention groups were compared to assess the impact of G-ANC on recognition of newborn danger signs and practices to keep newborns healthy compared to I-ANC. Results for data collected on postnatal visits for both mother and child at T2 for the intervention (G-ANC) and that for the control group (I-ANC) were compared to assess the effect of G-ANC on improving postnatal visits. Logistic regression adjusted for clustering was used to analyze changes over time and between the control and the intervention groups for the individual items. For the two scale items (danger/warning signs for newborn scale and healthy newborn scale), poisson regression models were used to assess the difference between the two groups over time, controlling for additional covariates such as age, whether this was the participants first pregnancy and highest level of education of the mother. All analyses was based on intention to treat, and Bonferroni correction was made where applicable for multiple comparisons.

### Ethical approval and ethical concerns

In keeping with 1964 Declaration of Helsinki, the study protocol was reviewed and approved by the Ghana Health Service Ethics Review Committee (GHS-ERC016/04/19) and the University of Michigan Health Sciences Behavioral Sciences Institutional Review Board (HUM-00161464). (ClinicalTrials.gov, NCT04033003) All participating individuals completed written informed consent. There were no participants younger than 16 years of age recruited, thus consent was obtained from each individual.

Ethical concerns related to this study included ensuring that women in group care received the same individual clinical care that women in individual antenatal care received, which we addressed by ensuring that each participant had a portion of their visit dedicated to individual clinical assessment prior to the start of group care. We also sought to ensure confidentiality of all data in several ways. First, clinical assessments were conducted separately from the group, and data were recorded privately. Second, all study-related data were collected privately by trained research assistants and entered directly into a password-protected tablet that recorded patient ID numbers instead of names. Only trained research personnel had access to the tablets and the data being collected. Finally, participants were reminded of the importance of privacy and confidentiality, and all participants were reminded at the beginning of every session that what was said during G-ANC by other participants should never be shared outside the group setting. Participants were not given an incentive to participate, however participants were given a newborn item such as a baby hat, baby blanket, or baby socks as a thank you for their participation.

## Results

Table 1 presents the demographic characteristic of the 1761 participants who were recruited into the study, which included 120 groups of 12 to 14 women of similar gestational ages upon presentation for ANC. This resulted in approximately 8 to 10 groups per facility. Most participants were less than 35 years old (84%), Only 20% of participants reported that they were pregnant with their first child. No significant differences were found between the G-ANC and I-ANC for the demographics considered. Since most of the participants (96%) were either married, cohabitating, or living together, no comparisons were made between the two groups.

**Table 1:** Demographics of the participant population (*N* = 1761)

Variable	Overall (*N* = 1761*) *N* (column percent)	I-ANC (*N* = 884) *N* (row percent)	G-ANC (*N* = 877) *N* (row percent)	*P*-value
Age Category (years)				
Less than 25	501 (28%)	266 (53%)	235 (47%)	0.193
25–34	987 (56%)	477 (48%)	510 (52%)	
35 or more	273 (16%)	141 (52%)	132 (48%)	
Maternal Education				
Primary	246 (14%)	120 (49%)	126 (51%)	0.6895
Middle/JHS/JSS	829 (49%)	429 (52%)	400 (48%)	
Secondary/SHS/Technical/Vocational	459 (27%)	223 (49%)	236 (51%)	
Tertiary	164 (10%)	83 (51%)	81 (49%)	
Partner Education				
Middle/JHS/JSS or less	666 (39%)	335 (50%)	331 (50%)	0.8525
Secondary	627 (37%)	306 (49%)	321 (51%)	
Tertiary	261 (16%)	126 (48%)	135 (52%)	
N/A, Unknown	137 (8%)	64 (47%)	73 (53%)	
Religion				
Christianity	1646 (93%)	835(50.4%)	811 (49.6%)	0.1185
Muslim	97 (6%)	39 (39%)	58 (61%)	
Other	18 (1%)	10 (56%)	8 (44%)	
First pregnancy				
No	1412 (80%)	703 (50%)	709 (50%)	0.4876
Yes	349 (20%)	181 (52%)	168 (48%)	
Location of Delivery				
Hospital/Polyclinic/Health Center	1711 (97%)	853 (50%)	858 (50%)	0.0904
Other	50 (3%)	31 (62%)	19 (38%)	
Continuous Items: Mean (SD)				
Maternal age	28.2 (5.8)	28.1 (6)	28.3 (5.6)	0.5042
Wealth index	6.8 (2.4)	6.9 (2.4)	6.9 (2.3)	0.6174
Number of previous pregnancies	3.5 (1.4)	3.5 (1.4)	3.5 (1.5)	0.7075

* This is the total sample size. If it doesn’t add across the demographics, then it is missing

Categorical variables were compared using chi-square test

Maternal age and wealth index tested using 2-sample t-test

*Number of previous pregnancies tested using Mann Whitney Wilcoxon test (non-parametric)*

^*a*^ *JHS = junior high school.* ^*b*^ *JSS = junior secondary school.* ^*c*^ *SHS = senior secondary school*

**Fig. 1:**
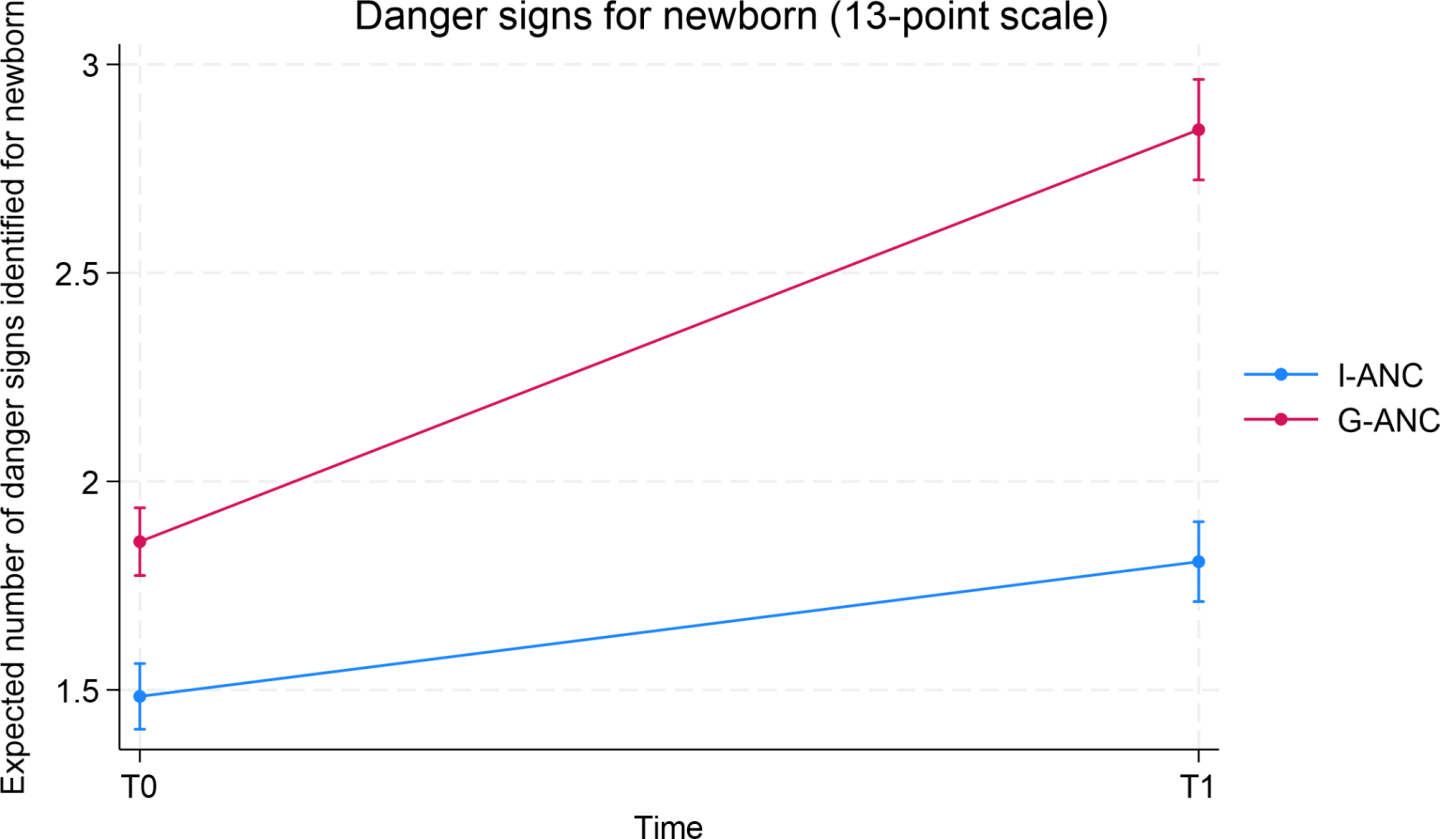
Danger signs for newborn by group and time

Table 2 summarizes the differences between I-ANC and G-ANC participants over time for the individual danger signs where a significant effect was found. Logistic regressions were run for the outcome variables listed in Table 2. The predicted probabilities are presented as percentages for the two groups and time periods. For all the items listed in Table 2, significant improvement was seen over time for both the groups, however, the improvement was significantly higher in G-ANC as compared to I-ANC in terms of their knowledge of danger signs for newborns at T0 and at T1. Table 3 summarizes the effect of the intervention (G-ANC vs. I-ANC) over time (T0 and T1) for the 13-point continuous scale of being able to recognize the danger/warning signs for newborn problems. (Each point on the scale represents a danger sign recognized.) Significant effects were observed for both groups over time, however improvement for the G-ANC was substantially higher than I-ANC over time (Fig. 1). In addition, education and whether this was the participant’s first pregnancy were also found to have significant effects. The higher the education, the better they were at recognizing danger signs for their newborn. Women who were not pregnant for the first time were better able to recognize danger signs than those who were pregnant for the first time.

**Table 2:** Recognition of Danger Signs for Newborn (individual items)

	% of women that responded “Yes”				
Item Description	I-ANC *n*(%)		G-ANC *n*(%)		Statistical significance for the two groups over time
	Time = T0	Time = T1	Time = T0	Time = T1	*P*-value
Too hot or too cold	481 (54.4)	405 (60.6)	536 (61.1)	471 (76.3)	0.0011
Convulsions/fits	28 (3.2)	32 (4.8)	33 (3.8)	77 (12.5)	0.0053
Feeding poorly or not at all	200 (22.6)	205 (30.7)	251 (28.6)	302 (49)	0.0015
Fast breathing or chest indrawn	40 (4.5)	30 (4.5)	37 (4.2)	97 (15.7)	< 0.0001
Umbilical cord stump is bleeding; has foul odor/redness around it	34 (3.9)	35 (5.2)	44 (5.0)	101 (16.4)	0.0003
Yellow skin, eyes, palms or soles of feet	133 (15.1)	154 (23.1)	156 (17.8)	213 (34.5)	0.0181
Red swollen eyes with pus	23 (2.6)	15 (2.3)	26 (3.0)	57 (9.2)	0.0008

**Table 3:** Recognition of Danger Signs for Newborn (13-point scale)

Predictor/covariate	Average Predicted Count	95% Confidence Interval	*P*-value
**Group**			< 0.0001
I-ANC (reference)	1.6	(1.6, 1.7)	
G-ANC	2.3	(2.2, 2.4)	
**Time**			< 0.0001
Time = 0 (Baseline) (reference)	1.7	(1.6, 1.7)	
Time = 1 (9-months gestation)	2.3	(2.2, 2.4)	
**Group effect over time**			< 0.0001
I-ANC Time = 0	1.5	(1.4, 1.6)	
I-ANC Time = 1	1.8	(1.7, 1.9)	
G-ANC Time = 0	1.9	(1.8, 1.9)	
G-ANC Time = 1	2.8	(2.7, 3.0)	
**Education**			< 0.0001
Primary (reference)	1.6	(1.5, 1.8)	
Middle	1.8	(1.8, 1.9)	
Secondary	2.1	(2.0, 2.2)	
Tertiary	2.5	(2.3, 2.7)	
**First pregnancy**			< 0.0001
No (reference)	2.0	(2.0, 2.1)	
Yes	1.6	(1.5, 1.8)	

**Fig. 2:**
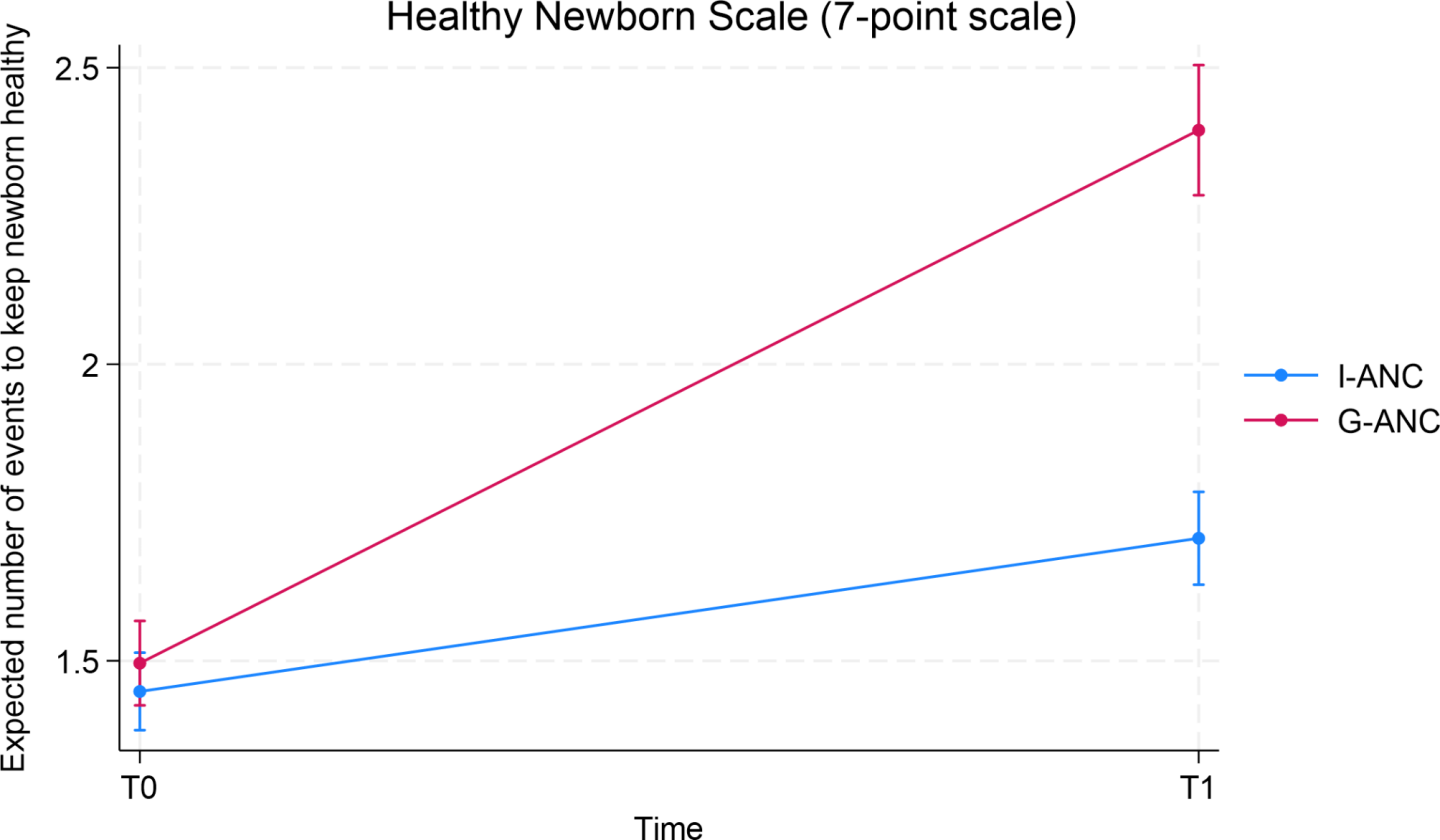
Healthy newborn scale by group and time

For all the items listed in Table 4, which illustrates recognition of factors needed to keep newborns healthy, the G-ANC group showed a significant increase compared to I-ANC over time. For the other items, no significant differences were observed between the two groups over time. Table 5 summarizes the effect of the intervention (G-ANC vs. I-ANC) over time (T0 and T1) for the 7-point healthy newborn scale. Conclusions from the model were similar to the 13-point scale of identifying danger signs for the newborn. Significant effects were observed for both the groups over time, however the improvement for the G-ANC was substantially higher than I-ANC over time (Fig. 2). In addition, education and whether this was the participants first pregnancy were also found to be significant. No differences were observed between primary and middle school educated women; Apart from that, the higher the education, the better they were at keeping their newborn healthy. Women who were not pregnant for the first time did better than than those who were pregnant for the first time.

**Table 4:** Recognition of factors needed to keep newborns healthy

	% of women that responded “Yes”				
Item Description	I-ANC *n*(%)		G-ANC *n*(%)		Statistical significance for the two groups over time
	Time = T0	Time = T1	Time = T0	Time = T1	*P*-value
Feed only breastmilk for 6 months	188 (21.3)	187 (28)	197 (22.5)	314 (50.9)	< 0.0001
Breastfeed often/whenever baby wants	479 (54.2)	436 (65.3)	403 (46)	398 (64.5)	0.0344
Wash hands with soap and water before handling baby	113 (12.8)	111 (16.6)	140 (16)	250 (40.5)	< 0.0001
Go for post-natal visit	141 (16)	107 (16)	145 (16.5)	148 (24)	0.0101

**Table 5:** Healthy newborn scale (7-point scale)

Predictor/covariate	Predicted number of events	95% Confidence Interval	*P*-value
**Group**			< 0.0001
I-ANC (reference)	1.6	(1.5, 1.6)	
G-ANC	1.9	(1.8, 1.94)	
**Time**			< 0.0001
Time = 0 (Baseline) (reference)	1.5	(1.4, 1.5)	
Time = 1 (9-months gestation)	2	(2.0, 2.1)	
**Group effect over time**			< 0.0001
I-ANC Time = 0	1.5	(1.4, 1.5)	
I-ANC Time = 1	1.7	(1.6, 1.8)	
G-ANC Time = 0	1.5	(1.4, 1.6)	
G-ANC Time = 1	2.4	(2.3, 2.5)	
**Education**			< 0.0001
Primary (reference)	1.6	(1.5, 1.7)	
Middle	1.6	(1.6, 1.7)	
Secondary	1.8	(1.7, 1.9)	
Tertiary	2.2	(2.0, 2.4)	
**First pregnancy**			< 0.0001
No (reference)	1.8	(1.7, 1.8)	
Yes	1.5	(1.4, 1.6)	

Table 6 demonstrates the differences between the I-ANC participants and the G-ANC participants in terms of postnatal care, as assessed at 6–12 weeks postpartum. The G-ANC participants were significantly less likely to report having a postnatal visit within 48 h of delivery than the I-ANC group. (67% vs., 76%, *p* = 0.0005) There was no difference between the groups in terms of postnatal visits at 6–7 days or 6 weeks. Overall, however, rates of postnatal care attendance for both groups were high at the 6-week postnatal visit, with the I-ANC at 91% and the G-ANC at 89%.

**Table 6:** Postnatal care (based on final T2 Data)

Label					
Continuous items	**I-ANC (** ***n*** ** = 596)**		**G-ANC (** ***n*** ** = 585)**		
					**P-value (T test)**
Categorical (Yes/No)	n	**Yes n(%)**	**n**	**Yes n(%)**	**P-value (Chi-sq. test)**
Postnatal visit at 2 days after delivery (48 h)	591	451 (76.3)	584	392 (67.1)	0.0005
Postnatal visit at 6–7 days	590	447 (75.8)	585	469 (80.2)	0.0684
Postnatal visit at 6 weeks	592	541 (91.4)	584	517 (88.5)	0.1029

Ref: prog2 analysis.sas

## Discussion

This study found that pregnant women who participated in G-ANC had significantly higher knowledge of newborn danger signs than those participating in I-ANC. Women in group care also had greater knowledge of strategies for keeping a newborn healthy when compared to women in individual care. However, group care was not associated with any increase in postnatal care seeking, with a slightly lower rate of postnatal care in the first 48 h among those who participated in group care.

Previous research on G-ANC has assessed newborn outcomes, such as birthweight, prematurity, small for gestational age, and neonatal intensive care unit admissions, in predominantly high-resource settings [18, 20, 28–33]. Recent work in Rwanda explored the impact of group care on length of gestation, finding no difference between groups in rates of prematurity [34]. This is the first study we are aware of that attempts to assess the impact of G-ANC on the process of improving newborn outcomes in a low-resource setting. Given the relative rarity of poor newborn outcomes, powering a study to detect differences in neonatal outcomes was beyond the scope of this research. Nonetheless, we were able to demonstrate that group care significantly improves mothers’ knowledge of newborn danger signs, which is the first step toward care-seeking for an ill newborn. Our study also found that mothers had better knowledge of how to keep their babies healthy. While this is not an assessment of behaviors, it is again an important indicator that women who participated in G-ANC were better positioned to promote healthy newborn habits.

This study has several important strengths. First, the cluster randomized controlled design was robust, ensuring that differences within individual facilities could be accounted for. In addition, the sample size of 1761 pregnant women across 120 groups was large enough to power rigorous analysis across several outcomes. This study also benefited from seasoned researchers with extensive field experience, hosted by the Dodowa Health Research Centre, which is home to a long-standing health and demographic surveillance site in southern Ghana [35]. This study was also subject to robust data checks and quality control, with regular interactions between the field team and those conducting data management activities. Finally, this study involved repeated trainings of both midwives and data collectors, ensuring fidelity to the intervention as well as fidelity to the data collection protocols [26]. 

Despite these strengths, there were some limitations to this study. First, the study was limited to the Eastern Region of Ghana, and it is possible findings might vary in other settings. Second, since the midwives in this study provided ANC for women in both group and individual antenatal care, it’s possible that some of the communication and information-sharing strategies they use during the group meetings were applied to the individual antenatal care. However, given the differences seen across group and individual care, we believe this limitation was effectively managed. Finally, due to the COVID-19 pandemic and other logistics, much of the postnatal data was collected via phone which might have yielded different responses when compared to face-to-face data collection.

The study also identifies a number of areas that require development and additional investigation. First, this study addresses an under-studied aspect of group antenatal care: the impact on newborns. However, this study was only able to assess maternal knowledge around danger signs and healthy newborn practices, not actual behavior. The only behavior we were able to record was postnatal care attendance, which notably, was lower among the group care mothers for the first (48-hour) post-natal visit and was no different for subsequent visits. This finding is counter-intuitive and warrants further investigation, although the overall prevalence of postnatal care was very high across both groups. Notably, this finding is consistent with other research in sub-Saharan Africa in which postnatal care rates were higher among the control group than among those participating in group antenatal care [34]. This raises questions about whether group care is creating a false sense of security that suggests to women they don’t need to go for postnatal care, having had such robust antenatal care. It is also possible that other factors are contributing to reduced postnatal care seeking for G-ANC participants in the days following birth, something that is worth exploring in greater depth than was possible in this research. Future research with larger sample sizes that could link knowledge with behaviors related to newborn care – especially follow-up care for unwell newborns in the months following birth – would be valuable to both researchers and practitioners.

Limitations notwithstanding, this research has several important implications. It is clear that G-ANC improves pregnant womens’ knowledge of newborn danger signs and healthy newborn habits when compared to individual care, suggesting that group care is one way to ensure that women are better prepared for the early days following birth. Given that much of the literature and programmatic emphasis surrounding G-ANC has focused on the benefits to the mother (e.g. health literacy, family planning uptake, ANC attendance [12, 33, 36]), our findings indicate that the impact for newborns may be just as important. As policy makers and program planners seek more interventions that can address the continuum from pregnancy through to the newborn and child years, group care is a viable option for expansion and further exploration. In addition, while newborn outcomes such as stillbirths, prematurity, early neonatal deaths, and NICU admissions are the indicators that often gain the most attention, knowing when to seek care for a sick newborn and knowing how to keep them healthy are critical to improving overall outcomes.

## Conclusion

In summary, this study provides important evidence of the value of G-ANC in terms of recognition of newborn danger signs and knowledge of healthy newborn practices. Further research that documents newborn care seeking in the face of illness and healthy habits adopted as a result of group care is warranted.

## Data Availability

Data are available through the University of Michigan’s permanent data repository, Deep Blue. Group Antenatal Care to Promote a Healthy Pregnancy and Optimize Maternal and NewbornOutcomes: A Cluster Randomized Controlled Trial (The GRAND project) https://doi.org/10.7302/d5ct-ne90(Data are available for download the the bottom of the screen).
